# Severe pre-eclampsia is associated with alterations in cytotrophoblasts of the smooth chorion

**DOI:** 10.1242/dev.146100

**Published:** 2017-03-01

**Authors:** Tamara Garrido-Gomez, Katherine Ona, Mirhan Kapidzic, Matthew Gormley, Carlos Simón, Olga Genbacev, Susan J. Fisher

**Affiliations:** 1 Center for Reproductive Sciences, University of California San Francisco, San Francisco, CA 94143, USA; 2 Department of Obstetrics, Gynecology, and Reproductive Sciences, University of California San Francisco, San Francisco, CA 94143, USA; 3 Fundación Instituto Valenciano de Infertilidad (FIVI), Instituto Universitario IVI, INCLIVA, Biomedical Research Institute, Valencia University, Valencia, 46010, Spain; 4 Fundación Igenomix, Valencia, 46980, Spain; 5 Department of Obstetrics and Gynecology, School of Medicine, Stanford University, CA 94305, USA; 6 The Eli & Edythe Broad Center for Regeneration Medicine and Stem Cell Research, University of California San Francisco, San Francisco, CA 94143, USA; 7 Department of Anatomy, University of California San Francisco, San Francisco, CA 94143, USA

**Keywords:** Pre-eclampsia, Preterm birth, Chorion, Cytotrophoblast, PAPPA1, Human

## Abstract

Pre-eclampsia (PE), which affects ∼8% of first pregnancies, is associated with faulty placentation. Extravillous cytotrophoblasts (CTBs) fail to differentiate properly, contributing to shallow uterine invasion and deficient spiral artery remodeling. We studied the effects of severe PE (sPE) on the smooth chorion portion of the fetal membranes. The results showed a significant expansion of the CTB layer. The cells displayed enhanced expression of stage-specific antigens that extravillous CTBs normally upregulate as they exit the placenta. Transcriptomics revealed the dysregulated expression of many genes (e.g. placental proteins, markers of oxidative stress). We confirmed an sPE-related increase in production of PAPPA1, which releases IGF1 from its binding protein. IGF1 enhanced proliferation of smooth chorion CTBs, a possible explanation for expansion of this layer, which may partially compensate for the placental deficits.

## Abstract (precis)

**Highlighted article:** Cytotrophoblasts (CTBs) within the smooth chorion of the human placenta are implicated in severe pre-eclampsia, undergoing significant expansion and changes in gene expression.

## INTRODUCTION

Human placentation involves a remarkable series of interactions between embryonic/fetal trophoblasts and maternal cells (Burton and Jauniaux, 2015; Maltepe and Fisher, 2015). In most of the villous placenta (chorion frondosum), cytotrophoblasts (CTBs) fuse to form a multinucleated epithelium that is perfused with maternal blood. Thus, they are ideally positioned to transport growth-promoting substances to the embryo/fetus, which they exchange for spent material. However, near the uterine wall, CTBs adopt a different fate. They leave the villi, forming columns of mononuclear cells, which anchor the placenta to the uterus, which they subsequently invade. The intricacies of CTB interactions with maternal cells that reside within the uterine wall involve many layers of cell-cell interactions. Within the interstitial compartment, they mingle with decidual and immune cells, eventually penetrating as far as the inner third of the myometrium. Invasive CTBs also remodel the uterine vasculature. They open up the spiral arteries, enabling placental perfusion, which substantially increases as they line and enlarge these vessels. They also breach uterine veins, which establishes venous return.

Defects in placentation are mechanistically related to several of the most clinically significant pregnancy complications. The CTB differentiation pathway that leads to uterine invasion appears to be particularly vulnerable, perhaps due to the unusual cell-cell interactions that occur, its explosive nature and the exceptional plasticity of the cells. Defects in this CTB subpopulation are associated with several pregnancy complications, but most consistently, pre-eclampsia (PE). In this syndrome, CTB invasion of the interstitial compartment is frequently shallow (Brosens et al., 1970). Similar patterns are observed during placentation in nonhuman primates, including the baboon and macaque (Enders et al., 2001), suggesting that this might not be a major determinant. Instead, failed vascular invasion is thought to be the crucial defect, a theory that is bolstered by the fact that restricting blood flow to the uterus and placenta creates some of the clinical signs of PE in animal models (Abitbol et al., 1977; Granger et al., 2006). Compared with normal pregnancy, many fewer spiral arteries show evidence of CTB remodeling, and the process is often less robust in those that do. Accordingly, they retain fundamental aspects of pre-pregnancy anatomy, which precludes carrying the amount of blood that the placenta needs to develop and function properly. Currently, development of PE is thought to involve a two-stage process in which abnormal placentation, the instigator, leads to a maternal inflammatory response (Redman and Sargent, 2005).

In comparison with the trophoblast components of the chorion frondosum, the CTBs that reside in the chorion laeve or smooth chorion (sch) have received little attention. schCTBs comprise the outer surface of the fetal membranes, external to the amnion and its stroma, which is shared with the smooth chorion. Here, this subpopulation of CTBs forms a second interface with the decidua, which they appear to invade, albeit more superficially than CTBs that emigrate from the chorion frondosum, which are found throughout the decidua basalis and inner third of the muscular portion of the uterine wall. Additionally, schCTBs do not invade uterine blood vessels. Thus, vascular remodeling is confined to the decidua basalis. Very little is known about the functions of these cells. A recent review suggested that they might play an active role, via their invasive activity, in fusion of the fetal membranes with the parietal decidua (Genbacev et al., 2015). This CTB subpopulation could also be involved in rupture of the fetal membranes at birth. In this regard, inducible nitric oxide synthase and cyclooxygenase-2 (also known as prostaglandin-endoperoxide synthase 2) contribute to the induction of apoptosis in these cells (Yuan et al., 2006, 2009), as does an imbalance in the production and elimination of reactive oxygen species (Yuan et al., 2008). Here, we tested the hypothesis that the defects in the chorion frondosum that are associated with PE extend to the smooth chorion. Surprisingly, the results showed changes consistent with the novel concept that, in this syndrome, the later population of CTBs expands, which could compensate for anatomical and functional deficits in the placenta.

## RESULTS

### The CTB layer of the smooth chorion expands in sPE

**Fig. 1.**
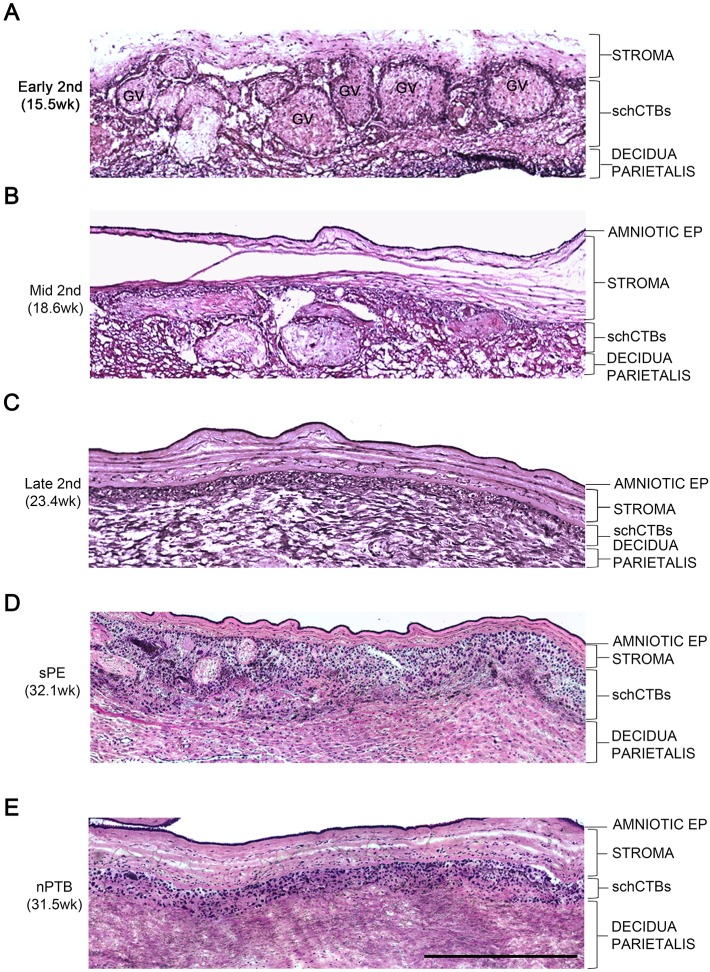
**The CTB layer of the smooth chorion expands in sPE.** Tissue sections from samples of this region, during the second trimester of normal pregnancy, and specimens from non-infected preterm birth (nPTB) and severe pre-eclampsia (sPE) were stained with H&E. (A) During early second trimester, the smooth chorion contained numerous ghost villi (GV). The CTB layer (schCTB) was disorganized, intermingling with the decidua. (B) As gestation proceeded, fewer GV were observed. (C) By the end of the second trimester, few (to no) GV were visible. The schCTBs became a defined layer adjacent to the decidua. (D) In sPE, the morphology of the smooth chorion resembled that of samples from the first half of the second trimester (compare A, B and D). (E) In contrast, the morphology of this region in nPTB was comparable to that of late second trimester specimens (compare C and E). In some cases, the stroma was more loosely organized than in the equivalent layer from sPE cases (compare D and E). *n*=5/group. EP, epithelium. Scale bar: 500 μm.

In cases in which the fetal membranes were intact, Hematoxylin and Eosin (H&E) staining revealed a single layer of amniotic epithelial (AMNION EP) cells that was connected to the smooth chorion via a shared stromal compartment. The outer CTB portion was in direct contact with the decidua parietalis. During early second trimester of normal pregnancy, the smooth chorion contained numerous villous remnants, termed ghost villi (GV) (Fig. 1A). The CTB layer (schCTB) was ill-defined and in some areas the fetal cells intermingled with the decidua. As gestation proceeded, the number of GV declined precipitously (Fig. 1B). By the end of the second trimester, few-to-none of these structures were visible. The schCTB layer had a more condensed appearance, and there was a defined border between the fetal cells and the decidua (Fig. 1C). It is likely that the observed changes are secondary to several factors. One is the loss of a direct blood supply. Another is establishment of the utero-placental circulation at the end of the first trimester of pregnancy, which results in a significant rise in oxygen tension. Placental perfusion is most robust at the center, creating oxidative stress at the margins (Burton et al., 2010).

We also analyzed tissue sections of the intact fetal membranes from five sPE and five preterm labor cases with no signs of infection (non-infected preterm birth, nPTB) (25-34 weeks). The maternal and neonatal characteristics are summarized in Table S1. Higher power magnifications of the photomicrographs revealed greater detail (Fig. 1) and lower power magnifications enabled assessment of nearly the entire membrane (Fig. S1). There were no notable differences between the appearances of the amniotic epithelium in the two pregnancy complications. In nPTB, some areas of the stroma appeared to be more loosely organized than in the equivalent layer from sPE cases (compare Fig. 1D,E). The major finding was the morphological resemblance of the smooth chorion from sPE cases to early and mid-second trimester samples (compare Fig. 1A,B,D). In addition to an expansion of the schCTB population, the layer was less organized and numerous GV were evident, which appeared with lower frequency in samples from nPTB cases that had morphological features of late second trimester samples (compare Fig. 1C,E). In some nPTB samples, the adjacent decidual layer was also somewhat disorganized. Thus, in sPE, the schCTB layer of the fetal membranes retained morphological features of the earlier gestation samples.

**Fig. 2.**
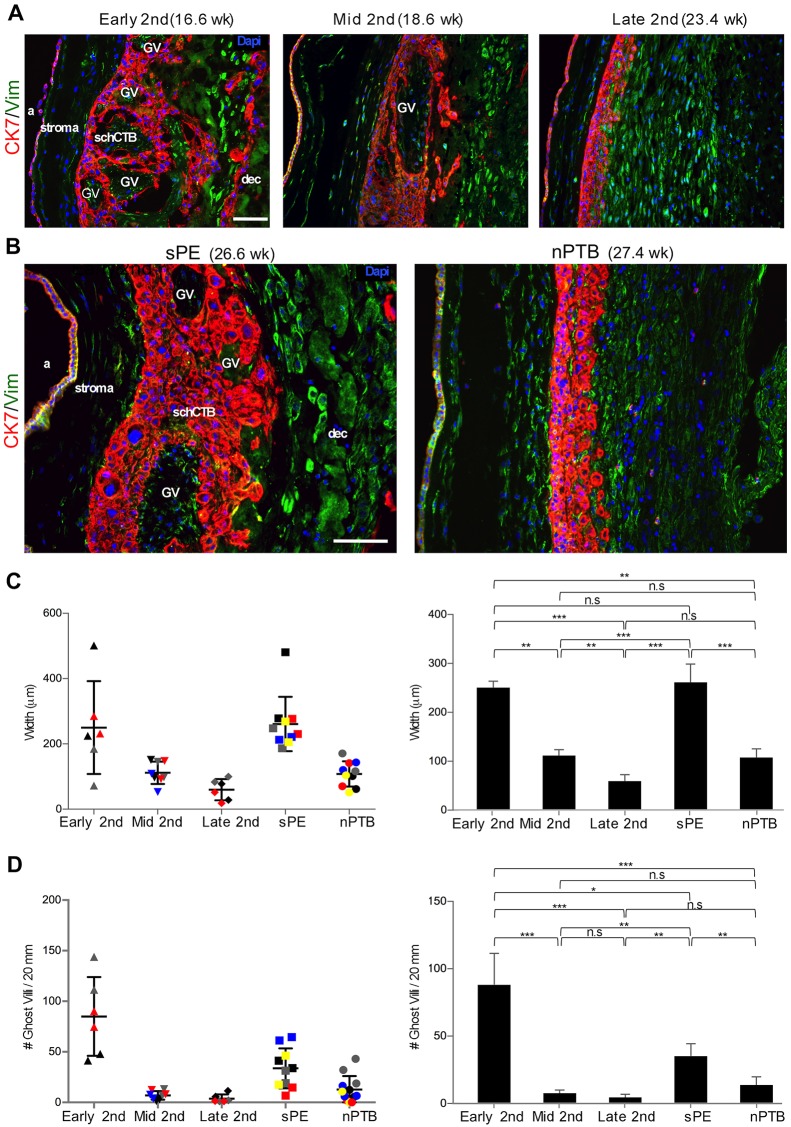
**Immunolocalization of cytokeratin confirmed expansion of the schCTB layer and retention of GV in sPE.** The samples were double immunostained. Anti-cytokeratin (CK7) labeled CTBs and amniotic epithelial cells. Anti-vimentin (Vim) reacted with the decidua, the stromal layer of the fetal membranes and, in some samples, the amniotic epithelium. (A) The results confirmed intermixing of the schCTB layer and decidua in early second trimester samples, the reduction in GV as pregnancy advanced, and the reorganization into a defined layer by late second trimester. (B) Cytokeratin immunolocalization also confirmed the morphological differences between the sPE and nPTB samples that were observed in the H&E-stained specimens (see Fig. 1D,E). (C) Quantification revealed an approximate 80% decrease in the width of the schCTB layer during the second trimester interval examined. The width of the schCTB layer in sPE was comparable to that of early second trimester specimens and nPTB samples were similar to the late second trimester samples. (D) Quantification revealed a dramatic reduction of GV during the second trimester of normal pregnancy. In sPE, the schCTB layer contained fewer GV than the early gestation samples, but more than in nPTB. *n*=5/group. In C and D, two areas of the same sample (individual samples denoted by different colors and symbols) were analyzed. *P<0.05; **P<0.01; ***P<0.001; n.s., not significant. a, amnion; dec, decidua. Scale bars: 100 μm.

To confirm and extend these findings, we double immunostained fetal membrane samples with anti-cytokeratin (CK), which labels CTBs and amniocytes, and anti-vimentin, which reacts with cells of the stromal layer of the membranes and the decidua (Fig. 2). As previously reported, amniocytes in some samples also gave a vimentin signal (Behzad et al., 1995). The results confirmed the intermixing of the schCTB layer and decidua in early second trimester samples and the reduction in GV as pregnancy neared the third trimester (Fig. 2A). CK immunolocalization confirmed the morphological differences between the sPE and nPTB samples (Fig. 2B) that were observed in the H&E-stained specimens (Fig. 1D,E). Next, we quantified two major features of the schCTB layer during the second trimester of normal pregnancy and in sPE versus nPTB. On average there was an approximate 80% decrease in the width of this layer during the second trimester (Fig. 2C). Measuring this parameter in sPE samples showed that the width of the schCTB layer was comparable to that of early second trimester specimens. In this regard, nPTB samples were similar to the mid and late second trimester specimens. As to GV, there was a dramatic reduction during the second trimester of normal pregnancy (Fig. 2D). In sPE, the schCTB layer contained approximately twice the number of GV compared with the nPTB samples, and the frequency was higher than in the mid and late second trimester specimens. Together, these results suggested that the schCTB layer of the fetal membranes in sPE retained the morphological features of the early second trimester samples. In nPTB, this layer had the morphology of late second trimester samples from normal pregnancy.

### sPE is associated with increased expression in schCTBs of a combination of villous and extravillous CTB stage-specific antigens

Next, we examined the expression of antigens that are misregulated in the basal plate region in pregnancies that are complicated by sPE. Specifically, invasive CTBs downregulate the expression of HLA-G (Lim et al., 1997), misexpress several integrins (Zhou et al., 1993) and upregulate E-cadherin (cadherin 1) (Zhou et al., 1997). These changes are associated with a failure of CTBs within the uterine wall to express vascular-type antigens (Zhou et al., 1997).

**Fig. 3.**
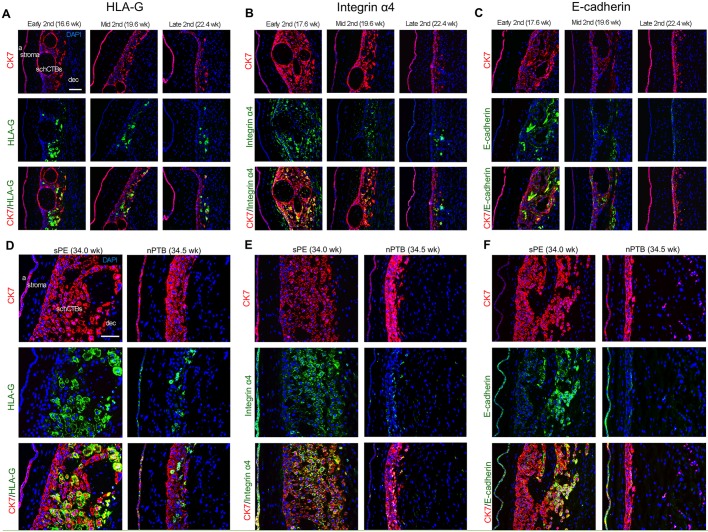
**In sPE, a subpopulation of schCTBs showed strong staining for stage-specific antigens, which invasive/extravillous CTBs upregulate as they emigrate from the placenta.** (A) In early second trimester of normal pregnancy, a subset of CTBs throughout the smooth chorion reacted with anti-HLA-G. By late second trimester, fewer cells immunostained. (B) The same general pattern was observed for anti-integrin α4 immunoreactivity. (C) Numerous schCTBs reacted with anti-E-cadherin in early second trimester. This subpopulation was largely absent in samples from the late second trimester of normal pregnancy. (D) Compared with second trimester and nPTB samples, the proportion of cells that reacted with anti-HLA-G was higher in sPE. (E,F) Expression of integrin α4 (E) and E-cadherin (F) was upregulated in sPE compared with nPTB. The images are representative of the analysis of a minimum of three sections from different samples (*n*=5). a, amnion; dec, decidua. Scale bars: 100 μm.

As to the smooth chorion in the second trimester of normal pregnancy, a subset of CTBs toward the decidual interface reacted with anti-HLA-G, anti-integrin α4 and anti-E-cadherin (Fig. 3A-C). In general, the number of cells that immunostained was reduced (HLA-G and integrin α4) or nearly absent (E-cadherin) by late second trimester. At this stage, immunopositive cells were most often found at the decidual boundary. We failed to detect expression in schCTBs of vascular-type molecules that extravillous CTBs express within the uterine wall and blood vessels – PECAM (platelet and endothelial cell adhesion molecule), VCAM (vascular cell adhesion molecule), VE-cadherin (cadherin 5) and VEGFA (vascular endothelial growth factor A) (data not shown). Thus, this subpopulation of CTBs has similarities with and differences from invasive extravillous cells.

In comparison to the second trimester of normal pregnancy, the proportion of cells that reacted with an antibody that specifically recognized HLA-G was higher in sPE (Fig. 3D). The fraction of cells that expressed this antigen in nPTB was similar to that observed in the late second trimester samples. With regard to the integrin α4 and E-cadherin expression patterns in the pregnancy complications that we studied, the major finding was upregulated expression in sPE compared with nPTB (Fig. 3E,F). For the three antigens whose expression we studied, the immunopositive cells were oriented toward the decidua rather than the amnion. As in the second trimester samples from normal pregnancies, no expression of the vascular-type antigens we assayed was detected in the pregnancy complication groups (data not shown). Together, these results suggested that schCTBs in sPE versus nPTB have more widespread expression of the stage-specific antigens that the extravillous subpopulation normally upregulate in columns and the superficial decidua.

### During the second trimester of normal pregnancy isolated schCTBs are more invasive than villous CTBs

**Fig. 4.**
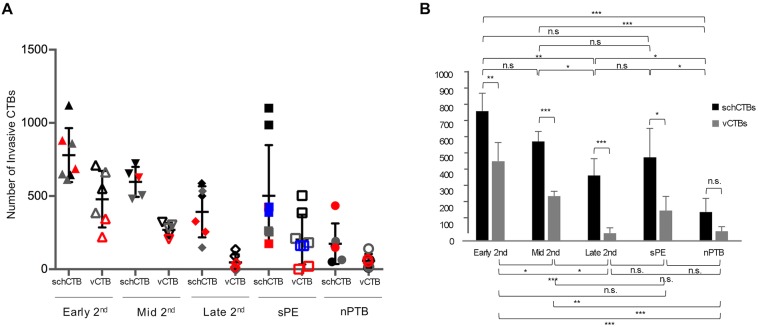
**Quantification of cytotrophoblast invasion: smooth chorion versus chorionic villi and sPE versus nPTB.** (A,B) Invasion was assayed using a Matrigel penetration assay in which cells are plated on Matrigel-coated filters and the number of cells that reached the filter underside were counted. The overall approach was to compare the behavior of cytotrophoblasts isolated from the two compartments. With the exception of nPTB samples, cytotrophoblasts from the smooth chorion (schCTB) were more invasive than their villous counterparts (vCTBs). During the second trimester of normal pregnancy, the invasiveness of CTBs isolated from both compartments decreased with advancing gestational age. sPE was associated with increased invasiveness of schCTBs compared with the comparable population of cells isolated from the placentas of nPTB cases such that the levels were similar to second trimester cells. *n*=3-4 CTB preparations isolated from different placentas per experimental group (denoted by colors). Samples were assayed in duplicate. *P<0.05, **P<0.01, ***P<0.001; n.s., not significant.

In these experiments, we quantified invasion by using a Matrigel penetration assay in which CTBs are plated on matrix-coated filters and the number of cells that reach the underside are counted. First, we compared the behavior of schCTBs and their villous (v) counterparts from the same donors. During the second trimester of normal pregnancy, CTBs from the smooth chorion were more invasive than vCTBs at all the gestational ages that were examined (Fig. 4A,B). The invasiveness of CTBs isolated from both compartments decreased during the second trimester.

### sPE is associated with increased schCTB invasiveness compared with nPTB

Next, we investigated whether the morphological and antigenic alterations that were observed in sPE were accompanied by functional changes. The maternal and infant characteristics of the samples that went into this analysis are included in Table S1. In sPE, schCTBs were once again more invasive than vCTBs and as compared with schCTBs and vCTBs in nPTB (Fig. 4A,B). Although variability impacted the statistical significance, CTBs from the sPE smooth chorion tended to mimic the invasion levels of the second trimester samples. In contrast, comparable levels of invasion were observed in the later second trimester, sPE and nPTB vCTB samples. Thus, these data suggested either an autocrine or a paracrine braking mechanism that restrains schCTB invasion *in vivo* and that sPE is associated with increased invasiveness of these cells, perhaps overcoming this barrier. We hypothesize that the sPE-related changes in the schCTBs subpopulation are, at least in part, a compensatory mechanism for functional deficits in vCTBs, particularly with regard to differentiation along the extravillous pathway.

### sPE impacts schCTB gene expression

To gain a better understanding of the molecular mechanisms behind the observed schCTB alterations in sPE, we used a laser capture microdissection approach to isolate this population of cells from the fetal membranes of sPE and nPTB pregnancies (*n*=4/group). The maternal and neonatal characteristics associated with the samples used for microarray analysis are summarized in Table S2. There were no differences in maternal characteristics other than elevated blood pressure and proteinuria in the sPE group. Neonatal weights were also lower in this group.

**Fig. 5.**
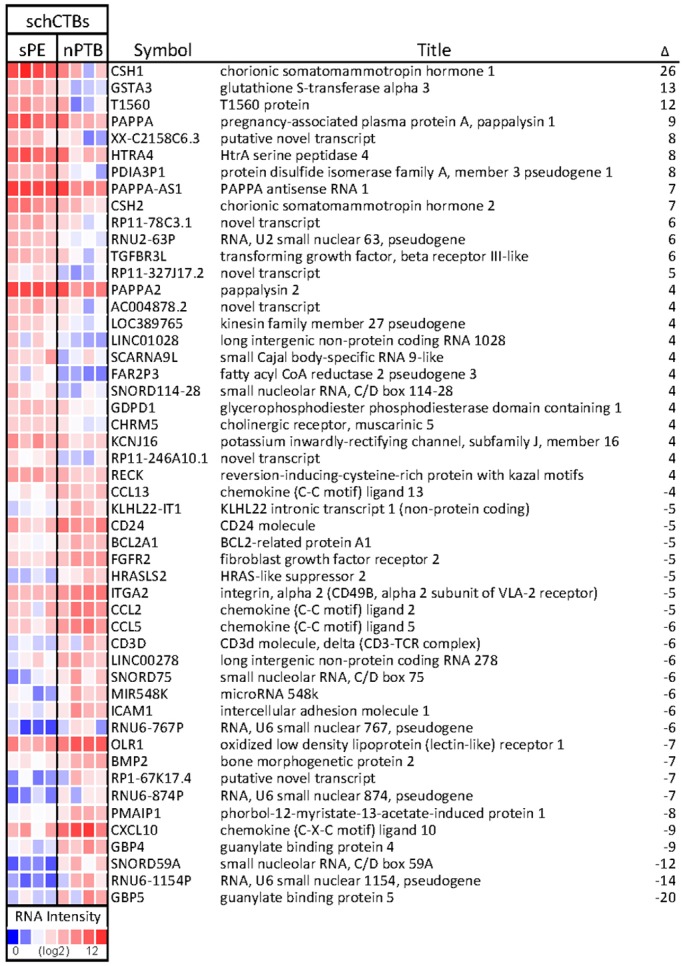
**Global transcriptional profiling of cytotrophoblasts from the smooth chorion revealed sPE-associated aberrations in gene expression.** The cells were isolated by laser capture microdissection. Then RNA was prepared and gene expression analyzed using an Affymetrix microarray platform. The relative expression levels of the 50 most highly differentially expressed genes (severe pre-eclampsia, sPE) versus non-infected preterm birth (nPTB) are shown as a heat map (red, upregulated; blue, downregulated). The fold changes are shown on the right (Δ). *n*=4 sPE samples and 4 nPTB samples. T1560 (LINC01602); KLHL22-IT1 (RNY1P9); LOC389765 (gene kinesin family member 27 pseudogene RP11-213G2.3).

Microarray analyses enabled a global comparison of the cells' gene expression patterns in the two conditions. In sPE schCTBs, 116 genes were upregulated (2-fold and higher) and 133 genes were downregulated (2-fold and higher) compared with nPTB (Fig. 5, 50 most highly differentially expressed; Fig. S2, complete list). The most highly upregulated genes were *CSH1* (also known as *HPL*; 26-fold), glutathione S-transferase alpha 3 (*GSTA3*; 13-fold) and *PAPPA1* (*PAPPA*; 9-fold). Two related molecules, PAPPA antisense RNA 1 and *PAPPA2*, were also upregulated (7-fold and 4-fold, respectively). Another placental lactogen, chorionic somatomammotropin hormone 2, was upregulated 7-fold. Thus, several placenta-specific proteins were modulated in sPE as was *GSTA3*, which functions in hormone production and detoxification. Upregulation of *TGFBR3L* (6-fold), which binds TGFβ family members via its heparan sulfate chains, suggests the possibility of enhanced growth factors signaling in sPE.

Conversely, we found upregulation, in nPTB, of immune molecules, guanylate binding proteins 5 and 4 (GBPs; 20-fold and 9-fold, respectively), *CXCL10* (9-fold), *ICAM1* (6-fold), *CD3D* (6-fold), *CCL5* (6-fold), *CCL2* (5-fold), *CD24* (5-fold) and *CCL13* (4-fold). As a group they have many interesting immune-related functions. GBP4 dampens interferon-induced viral responses (Hu et al., 2011) and GBP5 is an interferon-inducible inhibitor of viral infectivity including HIV (Krapp et al., 2016). These chemokine-related and adhesion molecules promote chemotaxis of monocytes/macrophages, T cells, NK cells and dendritic cells; T cell adhesion to endothelial cells; and angiogenesis. ICAM strengthens intercellular adhesion, and CD3D is a component of the T-cell receptor. These data suggested that inflammation and immune processes were activated in nPTB even when there were no clinical signs of overt infection. Other genes that were downregulated in schCTBs from sPE compared with nPTB (e.g. *BMP2*, 7-fold; *FGFR2*, 5-fold) might be related to aberrations in growth factor signaling.

We also compared gene expression of schCTBs in sPE and nPTB with the equivalent population of cells that were laser captured from fetal membranes, which were obtained during the second trimester of normal pregnancy (Fig. S3A). In general, the gene expression patterns of the nPTB samples were more similar to the second trimester schCTBs than were those of the equivalent sPE population. Hierarchical clustering failed to entirely separate the second trimester and nPTB samples. In contrast, the equivalent cells from sPE cases clustered together (Fig. S3B). Thus, although the schCTB layer of the smooth chorion had morphological features of early second trimester samples, global transcriptional profiling showed major differences at a molecular level.

In comparison with normal second trimester schCTBs, the mostly highly upregulated genes in sPE samples (Fig. S4) were *MT1H* (23-fold) and *MT1G* (13-fold), providing evidence of oxidative stress, which was consistent with the vascular constriction that was observed in the adjacent decidual compartment (data not shown). Corneodesmosin, which is related to cornification of epithelial layers, was also highly upregulated (10-fold) in sPE. Whether or not this is functionally related to the expansion of the schCTB layer in these cases remains to be determined. *PAPPA1* and *GSTA3* expression was also higher than in normal second trimester samples (8-fold and 4-fold, respectively). With regard to genes for which expression was downregulated in sPE versus second trimester samples, several chemokine family members were in this category, suggesting that their upregulation in nPTB might not be related to this condition, e.g. *CD24* (10-fold), *CXCL10* (6-fold) and *CCL2* (6-fold). Interestingly, expression of *NPPB* (11-fold), which acts to decrease blood pressure, was also reduced in sPE. Thus, immunolocalization of stage-specific antigens, invasion assays and gene expression data pointed to schCTB dysregulation in sPE.

We also compared gene expression of schCTBs in nPTB versus the equivalent population of cells that were laser captured from fetal membranes obtained during the second trimester of normal pregnancy (Fig. S5). The most highly upregulated genes included a serine peptidase inhibitor (*SPINK1*; 12-fold), *HLA-DR* (12-fold) and *BMP2* (7-fold). The genes downregulated to the greatest extent included histone *H2AM* (*HIST1H2AM*; 11-fold) and *CGA* (8-fold). Overall, small nuclear RNAs were highly differentially expressed in this dataset.

### Validation of the differentially expressed genes at the protein level

**Fig. 6.**
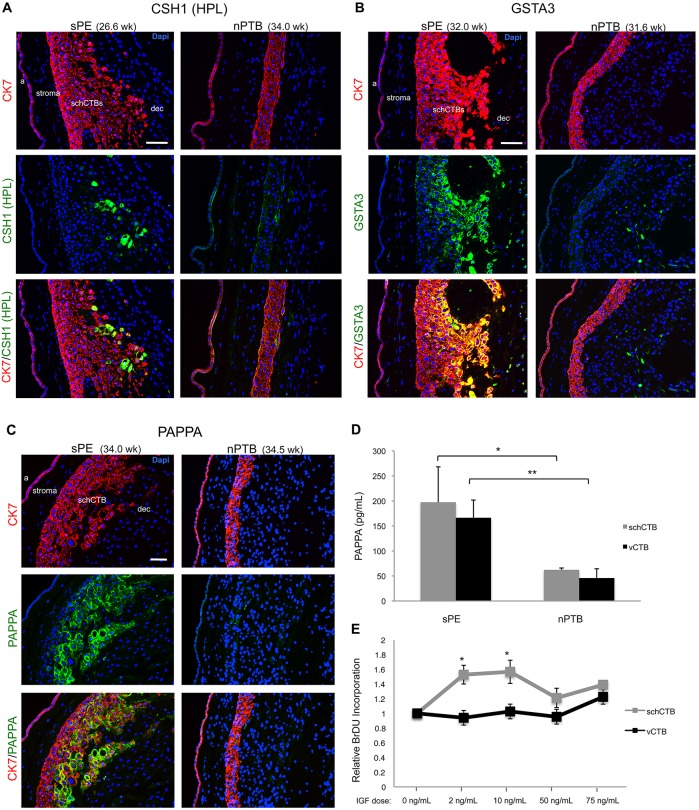
**Confirmation of the microarray results: protein level and functional data.** CSH1, GSTA3 and PAPPA1 immunoreactivity corroborated differential expression at the RNA level. The identity of cytotrophoblasts in the smooth chorion (schCTBs) was confirmed by cytokeratin (CK) expression. (A) Little to no signal for CSH1 was detected in cases of non-infected preterm birth (nPTB). In contrast, a subset of schCTBs interspersed among the immunonegative cells stained strongly with an antibody that recognized this molecule. (B) In sPE, the same pattern of differential expression was observed for GSTA3 except that immunoreactivity was more widespread among the schCTBs and the signal was associated with cells that were adjacent to the decidua parietalis. (C) Immunolocalization of PAPPA in the fetal membranes showed high CTB-associated immunoreactivity in sPE compared with largely background staining in nPTB. The images are representative of the analysis of a minimum of three sections from different areas of smooth chorion biopsies for each case (*n*=4/group). Cytokeratin (CK7) expression confirmed trophoblast identity and DAPI staining enabled visualization of nuclei. a, amnion; dec, decidua. Scale bars: 100 μm. (D) ELISA quantification of CTB PAPPA1 secretion into the culture medium. In sPE, vCTB and schCTB release of PAPPA1 significantly increased compared with the same subpopulations of cells in nPTB. *n*=3/group. (E) Isolated schCTBs and vCTBs (*n*=3 second trimester samples/group) were cultured in the presence of exogenous IGF1. Compared with baseline (no addition), IGF1 (2 and 10 ng) increased BrDU incorporation by schCTB, but not vCTBs. *P<0.05; **P<0.01.

Next, we investigated whether the highly upregulated mRNAs were accompanied by an equally dramatic increase in expression at the protein level. Accordingly, we immunolocalized CSH1 (HPL) in tissue sections of the fetal membranes from sPE and nPTB cases (*n*=4/group; Table S1). Little immunoreactivity was detected in the CTB layer of the smooth chorion from pregnancies that were complicated by nPTB. In contrast, sPE was associated with strong anti-CSH1 immunoreactivity in a subset of CTBs that tended to be located near the decidual junction (Fig. 6A). Essentially the same result was obtained for GSTA3. However, a greater proportion of the CTB population was immunopositive (Fig. 6B).

### PAPPA1-stimulated proliferation of schCTBs

Finally, we were interested in determining whether any of the upregulated molecules could be functionally related to the observed sPE-associated increase in the number of CTBs that resided within the smooth chorion. Given its ability to cleave IGFBP4 and -5, releasing the bound IGF1 (Gaidamauskas et al., 2013), we were interested in the expression and function of PAPPA1. Immunolocalization of this antigen in the fetal membranes showed high CTB-associated immunoreactivity in sPE compared with largely background staining in nPTB (Fig. 6C). Additionally, we assayed CTB secretion of PAPPA1 into the conditioned medium. In sPE, villous and schCTB release of PAPPA1 significantly increased compared with the same populations in nPTB (Fig. 6D). Thus, this pregnancy complication is associated with highly upregulated production and secretion of PAPPA1 by CTBs of the placenta and smooth chorion.

To determine the consequences, we isolated schCTBs and vCTBs (*n*=3 second trimester samples/group) and cultured them in the presence of exogenous IGF1. Compared with base-line (no addition), IGF1 (2 and 10 ng) increased bromodeoxyuridine (BrDU) incorporation by schCTB, but not vCTBs (Fig. 6E). Together, these data suggested that sPE-associated increases in PAPPA1 could be at least partially responsible for the observed expansion of the schCTB layer in this pregnancy complication. Alternatively, enhanced expression of PAPPA1 and its effects on CTB proliferation could be attributable to a delay or halting of differentiation such that, in sPE, the later gestation smooth chorion has features that are more typical of second trimester samples.

## DISCUSSION

It has been nearly 50 years since PE was linked to malformations of the maternal-fetal interface, in particular to deficient CTB remodeling of the uterine vasculature (Brosens et al., 1970). In normal pregnancy and in PE, very little is known about the other region where CTBs interface with the decidua – the smooth chorion. We studied this portion of the fetal membranes, during the second trimester of normal pregnancy, in relationship to sPE and nPTB. At a morphological level, two major features of the smooth chorion changed in the interval between early and late second trimester. The width of the CTB layer dramatically decreased along with the number of GV. In sPE, the smooth chorion retained characteristics of the early gestation normal samples – a wide CTB layer with numerous GV that were absent in the equivalent region of the nPTB samples, which resembled the morphology of the smooth chorion in the late second trimester of normal pregnancy.

At a molecular level, schCTBs were a distinct CTB subpopulation. Initially, we examined the cells' expression of stage-specific antigens that villous CTBs modulate as they exit the chorion frondosum and invade the decidua. In line with the fact that there was no morphological evidence of uterine blood vessel invasion, the cells did not express the vascular-type cell adhesion molecules that they normally upregulate within the uterine wall, including PECAM, VCAM, VE-cadherin and VEGFA (Zhou et al., 1997). They also did not express integrin α1, which plays an important role in invasion (Damsky et al., 1994). During the second trimester of normal pregnancy, schCTB immunostained for a combination of antigens that are expressed by villous and invasive/extravillous CTBs: E-cadherin, integrin α4 and HLA-G. In general, expression of these molecules in normal pregnancy tended to be more widespread throughout the CTB layer in early second trimester samples than in later gestation when fewer cells were antibody-reactive. In this regard, HLA-G and integrin α4 expression were confined to a subset of CTBs that were in direct contact with the decidua. Thus, CTBs of the smooth chorion had an antigenic profile that resembled cells in proximal regions of columns that have initiated differentiation along the invasive pathway.

Previous studies reported similarities (e.g. EGF receptor expression; Bulmer et al., 1989) and differences (e.g. lectin staining; Lalani et al., 1987) between schCTBs and vCTBs. Additionally, production of renin (Poisner et al., 1981; Symonds et al., 1968) and its substrate, angiotensinogen (Lenz et al., 1993), is concentrated in schCTBs. Yeh et al. (1989) described two mononuclear CTB populations in the chorion laeve. The first is vacuolated cells, with a clear cytoplasm that is rich in pinocytotic vesicles and lipid droplets. They react with antibodies that recognize human placental lactogen and placental alkaline phosphatase. The role of the latter molecule in absorption led the authors to suggest that these cells might play a role in this process. The second population of cells has an eosinophilic cytoplasm, which lacked vacuoles, and they express neither antigen. Both schCTB subtypes failed to express other molecules that are characteristic of vCTBs, including prolactin, pregnancy specific beta 1 glycoprotein and hCG beta. The antigen-positive and -negative cells that we describe, which did not clearly correspond to the either of these populations, might be a mixture of the two.

The significant morphological changes that were associated with the CTB layer of the smooth chorion in sPE were accompanied by alterations in the expression of E-cadherin, integrin α4 and HLA-G. Specifically, a large proportion of the CTBs expressed these antigens compared with the same subpopulation in either the late second trimester of normal pregnancy or in nPTB. Thus, sPE is associated with morphological and molecular alterations in the CTB layer of the smooth chorion that were reminiscent of the villous immaturity involving the chorion frondosum that was noted many years ago (Hustin et al., 1984).

The gestation-related and sPE-associated differences in the CTB subpopulation of the smooth chorion suggested the possibility of functional alterations. Accordingly, we investigated this possibility by assaying their invasive ability. With regard to normal pregnancy, second trimester schCTBs were more invasive than vCTBs isolated from the same placenta. Additionally, we observed a gestational-age related decline in invasiveness. We speculate that this surprising difference may be indicative of an active role for these cells in attaching the fetal membranes to the decidua parietalis. Whether the decidua capsularis is lost in the process or fuses with rest of the decidua during this process is uncertain (Benirschke et al., 2012). The mechanisms that restrain schCTB invasion *in vivo* are unknown, but could include enhanced expression of plasminogen activator inhibitor-1 (PAI-1 or SERPIN E1) by decidual cells in this region (Floridon et al., 2000).

Using the same experimental design, we investigated the impact of sPE on the invasiveness of schCTBs versus vCTBs. Bolstering our finding that CTBs from the chorion are more invasive than their villous counterparts, we observed the same phenomenon in sPE and nPTB samples. Furthermore, schCTBs from the placentas of sPE patients were significantly more invasive than those from the placentas of women who experienced preterm birth. We speculate that this might reflect an attempt to form more extensive interactions with maternal cells perhaps for absorptive purposes. The enhanced invasiveness of schCTBs in sPE together with the expansion of this layer described above could be in response to the pathological alterations of the chorion frondosum and invasive/extravillous CTBs that are associated with this pregnancy complication.

Global transcriptional profiling revealed the gene expression patterns of schCTBs in sPE compared with nPTB (and the second trimester of normal pregnancy). Previously, we used this approach to compare vCTB gene expression in the same pregnancy complications immediately after the cells were isolated and as they differentiated along the invasive pathway over 48 h in culture (Zhou et al., 2013). A comparison of the two datasets yielded new insights. First, the number and magnitude of changes in gene expression that were evident in the schCTB dataset were far greater than those observed for vCTBs. This finding suggested that sPE has very significant effects on the chorion laeve as well as the chorion frondosum. Second, there was no overlap in the schCTB and vCTB genes that were dysregulated in sPE and the same was true for nPTB. This result suggested that these are two very different CTB subpopulations in normal pregnancy and in their transcriptional responses to sPE and nPTB. For example, many of the PSGs were upregulated in vCTBs isolated from the placentas of sPE cases (Zhou et al., 2013). In contrast, only PSG11 was modulated (4-fold increase) in the equivalent schCTB dataset. Together, these findings suggested that schCTBs are active participants rather than passive bystanders to the placenta's role in pregnancy. In this regard it is interesting to note the geometry of the placenta and the very large surface area formed by the smooth chorion. Our results suggested the importance of considering this ‘second front’ where fetal and maternal cells directly interact, which, in addition to the chorion frondosum, could play an important role in governing pregnancy outcome.

As to the dysregulated expression of specific genes, *CSH1* was the most highly upregulated schCTB transcript in sPE. In vCTBs, another transcript from this locus, *GH2*, was the most highly expressed gene (Zhou et al., 2013). In contrast to both findings, analyses of placentas from sPE cases showed that transcription from the entire locus (*GH2*, *CSH1*, *CHS2* and *CSHL1*) was downregulated (Männik et al., 2012). CSH1, which is secreted into maternal blood, regulates maternal metabolic adaptation to pregnancy. Consistent with its role in promoting intrauterine growth, it is also found in the fetal circulation (Handwerger and Freemark, 2000). Our data suggested that schCTBs might be a particularly important source of the latter fraction. The *GSTA3* transcript was also highly upregulated. This enzyme, a member of the glutathione S transferase family, plays an important role in the generation of intermediate metabolites in the biosynthesis of progesterone and testosterone (Johansson and Mannervik, 2001). Its enhanced expression in sPE suggested that schCTBs upregulate steroid production. In a rat model of this pregnancy complication, treatment with 17-hydroxyprogesterone improved uterine perfusion (Amaral et al., 2015). Whether our observation is related in terms of increased *GSTA3* expression as being an attempt to upregulate blood flow to the placenta remains to be determined. Finally, schCTBs highly expressed *PAPPA1* (and *PAPPA2*) in sPE versus nPTB. This result was in accordance with previous reports of elevated levels of this proteinase in maternal serum and/or placentas from PE cases (Kramer et al., 2016). Our investigation of increased PAPPA1 production suggested an autocrine role in expansion of the schCTB layer in sPE. Also, these data might be indicative of the smooth chorion retaining features of a more immature state. Finally, this analysis highlighted genes that were upregulated in nPTB. They included *GBP4* and *-5*, which play important roles in inflammasome assembly and function (Shenoy et al., 2012; Tyrkalska et al., 2016), possible signals of the beginning of an inflammatory response despite the absence of clinical indicators.

We also compared gene expression of schCTBs in sPE and nPTB to schCTBs in the second trimester of normal pregnancy. In sPE, the highly upregulated genes included *MT1H* and *MT1G* (Fig. S4), metal-binding proteins that protect cells from oxidative stress (You et al., 2002). Thus, our results suggested that the smooth chorion is also affected by reduced uterine perfusion. We were surprised to find a strong upregulation of corneodesmosin in sPE. This molecule is the major component of desmosomes in the stratum corneum of the skin (Ishida-Yamamoto and Igawa, 2015). This raised the possibility that components of a desquamation-like process might be involved in separation of the smooth chorion from the uterus. In the skin, SPINK family members, serine proteinase inhibitors, prevent degradation of corneodesomosomes by inhibiting the actions of kallikreins. Interestingly, *SPINK1* was the most highly upregulated gene in schCTBs from nPTB versus the second trimester of normal pregnancy (Fig. S5). In the second trimester comparison, natriuretic peptide B was the most downregulated gene in sPE. Deletion in mice results in impaired cardiac remodeling (Tamura et al., 2000), raising the possibility that this peptide could be involved in aspects of maternal vascular responses to pregnancy that are impaired in sPE.

In summary, we studied the impact of sPE on the CTB layer of the smooth chorion. In comparison to the equivalent region in nPTB, we found a large number of differences at morphological and functional levels. We also found that sPE had very different effects on the chorion frondosum and the chorion laeve, possible evidence that the CTBs in the two areas are different subpopulations of cells. The results also raised the possibility of crosstalk between the two extra-embryonic regions that enabled the smooth chorion to compensate for deficits in the villous placenta. In this new light, we propose that CTBs that reside in the fetal membranes play a larger role in governing pregnancy outcome than was previously appreciated.

## MATERIALS AND METHODS

### Human tissue collection

The UCSF Institutional Review Board approved this study. Informed consent was obtained from all donors. Samples were collected within 1 h of the procedure, washed in PBS and kept in cytowash medium (DME/H-21 medium, 1% Glutamine Plus, 1% penicillin/streptomycin, 0.1% gentamycin) supplemented with 2.5% fetal bovine serum (FBS), placed on ice prior to processing. The second trimester samples were classified as early (15.5 to 17.6 weeks), mid (18 to 22.3 weeks) or late (22.4 to 24 weeks). The clinical characteristics of the sPE and nPTB pregnancies are summarized in Tables S1 and S2.

### Immunolocalization

Immunolocalization was carried out using previously published methods (Genbacev et al., 2016). The antibodies we employed are listed in Table S3 along with the working concentrations.

### Morphological evaluations of the smooth chorion

All biopsies (∼10×20-40 mm) were obtained either 5-10 cm (second trimester) or 10-15 cm (term) from the chorionic plate, from random areas of the smooth chorion (at least three per case). They were washed in PBS, fixed with 4% paraformaldehyde for 30 min and infiltrated with 5-15% sucrose. The samples were rolled end-to-end, embedded in OCT, and frozen in liquid nitrogen. Tissue sections from second trimester, sPE and nPTB samples (*n*=5/each group) were stained with H&E.

Immunolocalization of cytokeratin and vimentin was performed as described (Genbacev et al., 2016). Tilescan images of the entire tissue section were generated using a Leica DMI6000 B fluorescence microscope (Leica Microsystems) and LAS software. This method enabled evaluation of a large area of the tissue (∼10-30×1 mm wide). All measurements were performed using ImageJ (version 1.45s). Three sections from two areas of early second trimester (*n*=3), mid second trimester (*n*=4), late second trimester (*n*=3), sPE (*n*=5) or nPTB (*n*=5) specimens were examined. The width of the CTB layer was measured from the inner edge of CTBs adjacent to the stroma to the outer edge, which was adjacent to the decidua. Ten random measurements were made over the entire length of the samples. In parallel, GV in the same images were counted using ImageJ.

### Isolation of villous cytotrophoblasts

vCTBs were isolated from second and third trimester human placentas by published methods (Fisher et al., 1989; Kliman and Feinberg, 1990). Primary CTBs were from floating (second trimester and term) and anchoring (second trimester) villi, which were dissected from the placentas. The isolated cytotrophoblasts were ≥90% pure as shown by staining for cytokeratin 7.

### Isolation of schCTBs

After extensive washing with 1× PBS (Ca^2+^ and Mg^2+^ free) supplemented with 1% penicillin-streptomycin (100× stock solution; 10,000 units/ml penicillin and 10,000 µg/ml streptomycin), 0.003% fungizone (stock solution of 250 mg/ml) and 1% gentamicin, the amnion and smooth chorion were manually separated along their shared stromal plane. Next, the decidua parietalis was removed and discarded. The chorionic CTB layer was minced into small pieces (3-4 mm) and subjected to a series of enzyme digestion steps. The first incubation (15-30 min) was in PBS (10 ml/g of tissue) containing 3.5 mg collagenase, 1.2 mg DNase, 6.9 mg hyaluronidase and 10 mg bovine serum albumin. The supernatant was discarded. Then, the tissue was incubated for 20-40 min in PBS containing trypsin (6.9 mg trypsin, 20 mg EDTA, 12 mg DNase per 100 ml; tissue weight: dissociation buffer volume=1:8). Enzyme activity was stopped by adding an equal volume of cytowash containing 10% FBS. The cell suspension was filtered through a 70 μm sterile strainer and centrifuged at 1200 ***g*** for 7 min. A second collagenase digestion was performed by adding a 7× volume of the collagenase digestion buffer (see above), calculated on the basis of the weight of the cell pellet, followed by incubation for 15-30 min. The cell suspension was collected a second time by centrifugation. The cell pellets from the trypsin and second collagenase digestions were combined and purified over a Percoll gradient as described above for vCTBs.

### Invasion assays

CTB invasion was quantified by using our previously published methods (Genbacev et al., 2016). The entire experiment was repeated three to four times per group. Two inserts were evaluated for each sample type.

### Laser microdissection

We used laser capture microdissection to isolate schCTBs from normal second trimester, sPE or nPTB smooth chorion samples (*n*=4/each group). Portions of the smooth chorion, separated from the amnion as described above, were washed repeatedly in cold PBS to remove blood. Unfixed specimens were rolled end-to-end, placed in cryomolds containing OCT, frozen over a dry ice/ethanol slurry, and stored at −80°C. The blocks were sectioned at 20 μm using a Leica CM3050 cryostat, mounted on UV-treated PEN-membrane slides (ThermoFisher Scientific), and stored under ice prior to laser capture microdissection later that day.

Immediately prior to the procedure, sections were immersed in cold PBS until the OCT dissolved (∼1 min), dipped in 0.1% Toluidine Blue for 30 s, washed in cold PBS, dehydrated (30 s/treatment) in a graded ethanol series (75%, 95%, 100%), then rapidly dried with compressed nitrogen. All solutions were made with nuclease-free water.

schCTBs were laser dissected (Leica LMD 7000) and collected directly into RLT Plus Buffer (Qiagen RNeasy Plus Micro kit).

Total RNA was isolated according to the manufacturer's protocol and concentrations were measured photometrically (NanoDrop 2000c). RNA integrity was determined via microfluidic phoresis (Agilent Bioanalyzer 2100). The samples were stored at −80°C.

### Microarray analyses

Global gene profiling was accomplished using the GeneChip HuGene 2.0 ST array (Affymetrix). Sample processing and hybridization were carried out according to protocols that were devised by the UCSF Gladstone (NHLBI) Genomics Core Facility. Gene level expression data quality was confirmed, normalized (RMA) and summarized (Affymetrix Expression Console Software). Significant differential expression was determined by statistical analysis of false discovery rate (FDR<0.05) and absolute linear fold change >2 (Transcriptome Analysis Console Software).

### PAPPA1 ELISA

Sch- and vCTB-conditioned medium was isolated from sPE (*n*=3) and control nPTB (*n*=3) samples. The PAPPA1 concentrations of technical replicates were assayed by using a commercial ELISA kit (LifeSpan BioSciences) according to the manufacturer's instructions. Sample values (pg/ml) were extrapolated from the standard curve.

### IGF1-induced CTB proliferation

Freshly isolated second trimester vCTBs and schCTBs were plated at a density of 8400 cells per well of a 96-well plate coated with Matrigel diluted 1:2 in medium containing various concentrations of human recombinant IGF1 (291-G1-200, R&D Systems): 0, 2, 10, 50 and 75 ng/ml. BrDU (60 μM) was added simultaneously. After 16 h, BrDU incorporation was assessed according to the manufacturer's directions (ab126556, Abcam, UK). The entire experiment was repeated three times with duplicate or triplicate technical replicates.

### Statistical analysis

Data are shown as mean±s.e.m. Student's one-tailed *t*-distribution was used to compare the mean values among groups. Statistical significance was defined as *P*<0.05.
